# Plasma Exchange in Anti-Signal Recognition Particle Myopathy: A Systematic Review and Combined Analysis of Patient Individual Data

**DOI:** 10.3390/jpm14050461

**Published:** 2024-04-27

**Authors:** Pablo Martínez-Rodríguez, María Escribano-Iglesias, Ángel-P. Crisolino-Pozas, Noelia Cubino-Boveda, Miriam López-Parra, Miguel Marcos, Antonio-J. Chamorro

**Affiliations:** 1 Connective Tissue Diseases Unit, Department of Internal Medicine, University Hospital of Salamanca-IBSAL, 37001 Salamanca, Spain; pmartinez@usal.es (P.M.-R.); apcrisolino@saludcastillayleon.es (Á.-P.C.-P.); ncubino@saludcastillayleon.es (N.C.-B.); mmarcos@usal.es (M.M.); 2 Department of Medicine, Faculty of Medicine, University of Salamanca, 37001 Salamanca, Spain; 3 Department of Radiology, University Hospital of Salamanca-IBSAL, 37001 Salamanca, Spain; mescribanoi@saludcastillayleon.es; 4 Department of Hematology, University Hospital of Salamanca-IBSAL, 37001 Salamanca, Spain; mloparra@saludcastillayleon.es

**Keywords:** anti-SRP myopathy, immune-mediated inflammatory myopathy, plasma exchange, plasmapheresis

## Abstract

Anti-signal recognition particle myopathy (anti-SRP myopathy) is a rare subtype of immune-mediated inflammatory myopathy characterized by muscle weakness and anti-SRP autoantibodies. Although plasma exchange (PE) is used in severe cases, its role remains unclear. A systematic review was conducted following PRISMA guidelines, identifying 23 patients with anti-SRP myopathy treated with PE. Data on demographics, clinical features, laboratory findings, treatments, and outcomes were analyzed combining individual patient data if available. Sixteen (69.6%) patients were male, with muscle weakness as the predominant symptom in 100% of cases. After PE, most patients showed improvement in symptoms, and the proportion of patients with muscle weakness was reduced (*p* = 0.001). Relapse occurred in 17.4% of the cases. The incidence of adverse events was low (8.7%). Despite limitations, including a small sample size and heterogeneous data, our systematic review suggests that PE may be effective in inducing remission and controlling symptoms in anti-SRP myopathy, particularly in severe cases. Since evidence on PE in anti-SRP myopathy is limited, further research, including prospective multicenter studies, is warranted to understand better its efficacy and safety and establish its role in treatment algorithms.

## 1. Introduction

Anti-signal recognition particle myopathy (anti-SRP myopathy) is a rare subgroup of immune-mediated inflammatory myopathy (IMM), also known as immune-mediated necrotizing myopathy (IMNM), characterized by muscle biopsy findings of necrosis with little or no lymphocytic infiltration [1,2,3,4,5,6,7]. The disease is distinguished by the presence of autoantibodies. To date, the following two different autoantibodies have been described in IMNM: antibodies against signal recognition particles (anti-SRP) and antibodies against 3-hydroxy-3-methylglutaryl-coenzyme A reductase protein (anti-HMGCR). However, autoantibodies are not detected in up to 12% of patients, thus establishing a third group of seronegative patients [2,5,6].

The pathogenesis of the disease remains unknown, although there are several theories, none of which have managed to stand out from the others. The higher number of cases diagnosed in autumn suggests the possibility of an infectious or environmental trigger [5]. Immunogenic predisposition has been described for mutations in some DR alleles of the major histocompatibility complex, although in the Caucasian population, it has not been associated with any genetic markers to date [1]. Unlike anti-HMGCR myopathy, anti-SRP myopathy does not relate to cancer [4].

Regarding the prevalence of the disease, a review of 46 publications from 1966 to 2013 estimated the overall prevalence of idiopathic inflammatory myopathies to be between 2.4 and 33.8 cases per 100,000 population. Many patients are misdiagnosed with polymyositis, making it difficult to determine the true proportion of patients with IMNM. About 5–15% of patients diagnosed with idiopathic inflammatory myopathy (IIM) have anti-SRP antibodies [1].

The average age of onset ranges from 40 to 50 years, although it can occur during childhood. Both children and adults have a higher incidence rate in females and, unlike other myopathies, no clear predominance of the African population over the Caucasian or Asian population has been observed [4].

The initial symptom is typically acute or subacute proximal muscle weakness, which may be the only manifestation of the disease. Other symptoms such as dysphagia, myalgia or weight loss may coincide with disease flares [1,4,6]. Interstitial lung disease is the most common extramuscular manifestation, occurring in 23–38% of cases, although it may be asymptomatic or have normal respiratory function tests [1,7,8].

In 2017, the European League Against Rheumatism/American College of Rheumatology (EULAR/ACL) established a score and classification tree for patients with suspected IIM [9]. The presence of anti-SRP autoantibodies in patients with clinical or histological features of IMNM characterizes the diagnosis of anti-SRP myopathy. In fact, due to the specificity of these autoantibodies, the tandem of elevated creatin kinase (CK) levels and autoantibodies makes it possible to reach a diagnosis without the need for muscle biopsy, as these patients may have higher CK levels than those with other myopathies, with an average of approximately 4700 IU/L, according to some authors [1,4,5,6,8,10,11].

CK is also the most reliable marker for relapse, although in severely affected patients, muscle tissue may have been replaced by fatty tissue and fibrosis [12]. In cases where CK levels do not make it possible to assess disease activity, MRI can be useful. Both acute involvement and chronic muscle damage can be appreciated as this tool is mainly used to assess global damage and follow-up. However, it does not allow for the diagnosis of the disease, as it does not discriminate between the different subtypes of IMNM [1,4]. Electromyography is another test frequently used in the study of myopathies, which helps in the differential diagnosis of other diseases. It can confirm the pattern of myopathic involvement, i.e., polyphasic motor unit potentials of decreased duration and amplitude upon voluntary activation and increased spontaneous activity [13]. It can act as a guide to select where to perform a biopsy, together with MRI. Typical histopathological findings include necrosis of muscle fibers and regenerative foci, but mild or absent inflammatory infiltrates are the main difference with other inflammatory myopathies [4,12].

The treatment of anti-SRP myopathy focuses on immunosuppressants. Corticosteroids (CC) are the first-line agents, administered orally or intravenously, with an initial dose of 1 mg/kg/day prednisone and the subsequent addition of a corticosteroid-sparing agent, such as methotrexate (MTX). Rituximab (RTX) in two doses of 750 mg/m2 (maximum 1 g) on day 1 and day 7 or 15 has been shown to be effective as a first-line treatment, either as a replacement for methotrexate or in combination with corticosteroids [1,6,11,14]. The use of intravenous immunoglobulins (IVIGs) at a dose of 2 g/kg/month is recommended for patients with torpid evolution, and the assessment of plasma exchange (PE) in the management of patients with anti-SRP myopathy contrasts with the scarcity of evidence-based studies, as it should be considered a potential treatment option in critically ill patients [1,4,10,14]. Therefore, we conducted a systematic review of the literature to investigate the use of PE.

## 2. Materials and Methods

We conducted a systematic review according to the PRISMA guidelines. We used the following search terms: “anti-signal recognition particle”, “anti-SRP”, “antiSRP”, “plasmapheresis” and “plasma exchange”. References of the relevant articles retrieved from the initial search were manually identified and also reviewed. 

### 2.1. Inclusion Criteria

Patients with available data from published reports were included, regardless of publication date, if they met the diagnostic criteria for anti-SRP autoantibody-associated myopathy according to the 224th ENMC International Workshop, and if they had received treatment for PE at any point of the disease. Clinical manifestations and therapeutic information were also recorded. Patients diagnosed with other necrotic myopathies were excluded from the analysis.

### 2.2. Data Sources

The following databases were exhaustively reviewed: PubMed, Web of Science, Embase, and Scopus for articles with information on case reports and case series of patients with anti-SRP myopathy and PE treatment until February 2024, without language restrictions.

### 2.3. Selection and Data Collection

Data were recorded and extracted as individual patient data, if available, including age, sex, race, clinical features (pulmonary, dermatological, vascular, articular, and cardiac involvement), laboratory values (CK, anti-SRP autoantibodies, and anti-Ro52 antibodies, if available), initial and posterior treatment, relapse, and time to relapse. In the absence of uniform criteria for defining remission after flares, disease activity was categorized as “complete remission”, “clinical improvement” or “null response” based on the recovery, persistence, or worsening of analytical and clinical manifestations, respectively.

### 2.4. Statistical Analysis

A database of the extracted data was generated and analyzed using SPSS v28.0. The distribution of continuous variables was described as the number of observations with means and standard deviations. Qualitative values were reported as the number and percentage of patients. McNemar’s test was used to compare the trends between groups of qualitative variables, with a significance level of alpha = 0.05.

## 3. Results

### 3.1. Study Selection

**Figure 1:**
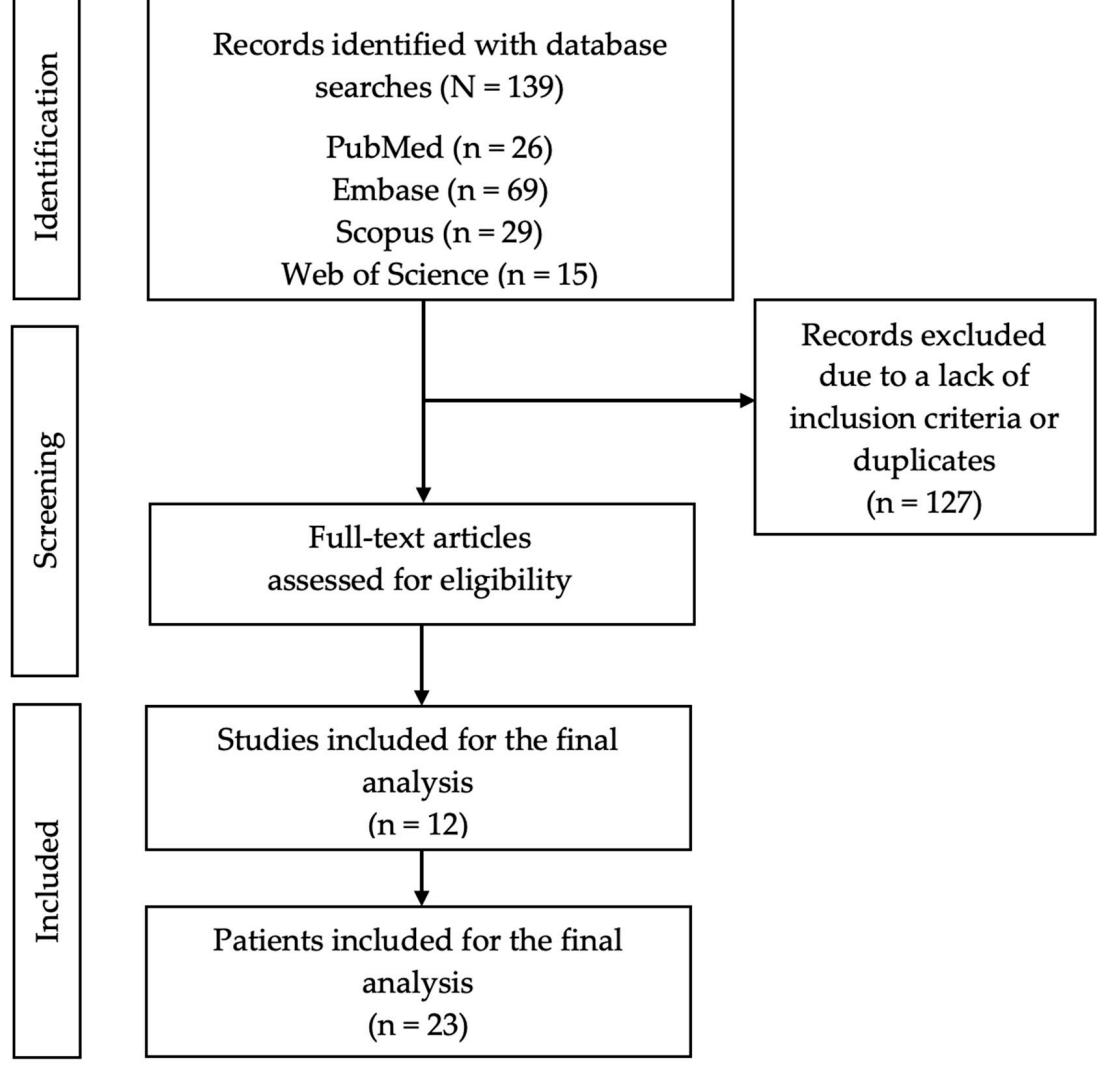
Flowchart of the review process.

Figure 1 displays the PRISMA diagram, which provides an overview of the search, along with an explanation of the exclusions.

### 3.2. Study Characteristics

In February 2024, a search for anti-SRP myopathy cases treated with PE returned 139 articles, of which 127 were excluded. Of the remaining 12 articles, clinical data were extracted for 23 patients who met the inclusion criteria (Figure 1) [15,16,17,18,19,20,21,22,23,24,25,26].

### 3.3. Epidemiological Findings

Of these patients, 7 (30.4%) were female and 16 (69.6%) were male, with a mean age at presentation of 42.4 years (SD = 20.8). There were only three patients under 18 years of age (15, 15 and 16 years). The majority of patients, 14 (60.9%), were of unknown ethnicity, while only 3 (13%) were Caucasian (Table 1).

### 3.4. Clinical Features

The most common initial symptom was muscle weakness, which was present in all 23 (100%) patients, followed by dysphagia in 7 (30.4%) patients. The most prevalent extramuscular manifestations were pulmonary and dermatological (30.4%), followed by cardiological (8.7%). Only one (4.3%) patient was diagnosed with neoplastic disease. Anti-SRP antibodies were present in almost all patients (95.7%) at the onset of the disease, although they were only analyzed after disease remission in four (17.4%) patients. Of these, three (75%) were positive. Anti-Ro52 antibodies were present in 12 (52.2%) patients at disease onset, followed by anti-MDA5 in 2 (8.7%) patients. The mean CK level at the time of diagnosis was 8586.6 IU/L (SD = 886.2) and, after treatment, the levels decreased to 573.3 IU/L (SD = 533.5). The mean aldolase and lactate dehydrogenase (LDH) levels before treatment were 141.9 IU/L (SD = 84.5) and 847.4 IU/L (SD = 311), respectively. Muscle biopsy was performed in 12 (52.2%) patients, and the results were consistent with those of anti-SRP myopathy in all of them. A neuromuscular study was conducted in 19 (82.6%) patients, which was also compatible with anti-SRP myopathy in all of them.

Several patients showed positive outcomes, with 10 (43.5%) achieving complete remission and an equal number experiencing clinical improvement. In contrast, two (8.7%) patients did not show improvement after PE. In terms of clinical response after PE treatment, only nine (45%) patients experienced ongoing muscle weakness, which reached statistical significance, while their other symptoms were resolved (Table 2).

As shown in Table 1, all patients had received CC therapy, and four of them (17.4%) had received intravenous pulses. IVIGs were administered to 13 (56.5%) patients. Azathioprine (AZT) was used in five (21.7%) patients, while tacrolimus (Tac) and cyclophosphamide (CYC) were used in four (17.4%) patients. RTX was used in three (13%) patients.

Among the patients with available data, only IVIGs showed statistically significant differences with respect to their use before and after PE (Table 3).

In terms of adverse effects (Table 1), one patient had an allergic reaction, whereas another patient discontinued PE after the fifth session because of mesenteric ischemia. Relapse occurred in four (17.4%) patients before PE, with 3.25 (SD = 1.9) episodes per patient experiencing relapse. The time until remission was 31.6 (SD = 63) months. Finally, three (13%) patients died.

## 4. Discussion

To the best of our knowledge, this is the first systematic review to examine PE as a treatment option for anti-SRP myopathy. In this regard, this work revealed a lack of uniformity in the management of this illness and the use of PE, which is consistent with the lack of an established position regarding this treatment in the management algorithms for IMNM, beyond its recommendation in severely affected patients [1,14].

In any case, our main finding is the potential efficacy of PE, since a significant proportion of patients experienced relief of muscle weakness and all patients had an improvement in the remainder of symptoms (Table 1 and Table 2). Although we lack high-quality evidence due to the difficulty in conducting controlled trials in critically ill patients and in diseases with such a low prevalence, in other diseases in which PE is an important part of the therapeutic algorithm, such as thrombotic thrombocytopenic purpura, myasthenia gravis, or other neurological disorders, multiple studies have also highlighted the efficacy of PE in improving clinical manifestations in the short term compared with other therapeutic options. Previous research has also noted a lower prevalence of adverse effects with PE than with other treatments, such as IVIGs, although more serious complications can occur [27,28,29]. Therefore, further studies are needed to collect more detailed safety information.

Although the number of patients was small, our systematic review provides a deeper understanding of this treatment considering previous studies on this topic (mostly small case series or single-case reports). Median age was comparable to previous findings in anti-SRP myopathy, but our study included a larger proportion of male participants than female participants, which contrasts with the higher prevalence of anti-SRP myopathy in women [1,4,5,11,14,30]. Although this may be an incidental finding owing to the small number of patients, patients receiving PE may represent a subset of patients with specific characteristics such as higher severity or lack of response to other therapies. 

Regarding clinical features, patients showed a moderate or severe presentation since 78.3% presented with muscle weakness with at least other symptoms. The proportion of specific symptoms was consistent with previous reports, and patients did not exhibit a higher prevalence of more severe clinical features, such as dysphagia or dyspnea [1,4,5,7]. With regard to the severity of the disease and the use of PE as a second- or third-line drug, more than half of the patients in our sample used at least two drugs prior to PE treatment, and around one-third used three or more different previous treatments (Table 1). It is worth noting that patients did not receive IVIGs after PE treatment, which reached statistical significance when compared with IVIG treatment before PE (Table 3). This may be explained by the fact that IVIGs may have been used for quick relief in a manner analogous to other IMNM such as in anti-HMGCR myopathy [10,11,31].

Another important finding is the presence of specific autoantibodies, which may play a fundamental role in pathogenesis [32]. As anticipated, almost all patients tested positive for anti-SRP antibodies at disease onset. Notably, nearly half of the patients with available data had positive anti-Ro52 test results. These autoantibodies are associated with a higher incidence of interstitial lung disease and elevated mortality in patients with myositis [33,34]. There was no other anti-Ro autoantibody type registered by authors, as other autoantibodies related to myopathy like anti-MDA5, anti-NXP2, anti-PM-Scl 75 and anti-Mi2 alpha were less prevalent. More research is needed to determine if there is a correlation between the presence of these autoantibodies or others found in our sample, such as anti-MDA5, anti-NXP2, or anti-Mi2 alpha, and a worse prognosis. This may potentially help us to identify patients with a higher risk of severe disease and to use PE as an early intervention rather than limiting it to patients with severe symptoms.

The variability in clinical presentation and potential severity of illness is underscored by the number of patients experiencing relapses and the variability in the disease-free interval (Table 1). These findings are only partly attributable to differences in the duration of the therapeutic effect of PE, but they reinforce the need for prognostic markers to identify patients at risk of severe disease. Particularly in children, a higher risk of relapse has been described, although the three patients under 18 years of age in our sample did not present any. This event was described by Kawabata et al., highlighting the importance of early identification of the disease and aggressive treatment from the onset, which may explain our observed results [16]. It is important to highlight that, since the use of PE in anti-SRP myopathy is not standardized, there is great variability in its use depending on the patient’s clinical condition and the experience of the treating team, from single treatment sessions to weekly cycles for years [19,22]. Further studies are needed to explore these and other aspects, since our systematic review is limited by the small number of patients available, the heterogeneous nature of data collection, the scarcity of information in some reports, and the lack of follow-up.

## 5. Conclusions

In conclusion, the limitations of this systematic review, including the scarcity of available data and lack of randomized studies in patients with anti-SRP myopathy, make it difficult to establish strong recommendations regarding the use of PE for treating anti-SRP myopathy. However, previous reports and our systematic review suggest that PE may be useful for inducing remission and controlling disease symptoms. Given the low prevalence of the disease, prospective multicenter studies may be a more feasible approach than controlled clinical trials.

## Figures and Tables

**Table 1:** Clinical, laboratory, histopathologic, therapeutic, and disease course data of 23 anti-SRP patients treated with plasma exchange.

	Current review
	PE
	*n* = 23 (%)
Sex female/male	7 (30.4)/16 (69.6)
Age at diagnosis, mean years (SD)	
Males	50.1 (22.1)
Females	38.9 (19.9)
Total	42.4 (20.8)
Race	
Asiatic	2 (8.7)
Caucasian	3 (13)
Afro-American	2 (8.7)
African	2 (8.7)
Not available	14 (60.9)
Initial symptoms	
Muscle weakness	23 (100)
Myalgia	6 (26.1)
Dysphagia	7 (30.4)
Anorexia	1 (4.3)
Dyspnea	3 (13)
Asthenia	2 (8.7)
Two or more	18 (78.3)
extramuscular manifestations at disease onset	
Yes/No	11 (47.8)/12 (52.2)
Pulmonary	7 (30.4)
Dermatological	7 (30.4)
Raynaud phenomenon	1 (4.3)
Cardiological	2 (8.7)
Joints	0
Two or more	5 (45.5)
Co-diagnosis with other disease	
Yes/No/Unknown	1 (4.3)/22 (95.7)
Neoplasic	1 (100)
Autoimmune	0
Anti-SRP antibodies documented	
At disease onset	
Positive	22 (95.7)
Negative	1 (4.3)
Not available	0
After treatment	
Positive	3 (13)
Negative	1 (4.3)
Not available	19 (82.6)
Anti-Ro52 antibodies at disease onset	
Positive	8 (34.8)
Negative	9 (39.1)
Not available	6 (26.1)
Other auto-antibodies	
Anti-MDA5	2 (8.7)
Anti-NXP2	1 (4.3)
Anti-PM-Scl 75	1 (4.3)
Anti-Mi2 alpha	1 (4.3)
Anti-Yo	1 (4.3)
Anti-dsDNA	1 (4.3)
Creatine kinase (IU/L)	
Before treatment; SD	8586.6; 886.2
After treatment; SD	573.3; 533.5
Aldolase (IU/L)	
Before treatment; SD	141.9; 84.5
After treatment; SD	Not available
Lactate dehydrogenase (IU/L)	
Before treatment; SD	847.4; 311
After treatment; SD	Not available
Muscle biopsy	
Yes/No	12 (52.2)/11 (47.8)
Compatible/Not compatible	12 (100)/0
Neuromuscular study	
Yes/No/Unknown	19 (82.6)/2 (8.7)/2 (8.7)
Compatible/Not compatible	19 (100)/0
Treatment before PE	
Glucocorticoids (any)	22 (95.7)
Intravenous pulses	4 (17.4)
Methotrexate	3 (13)
Azathioprine	5 (21.7)
Tacrolimus	4 (17.4)
Cyclosporine	1 (4.3)
Mofetil mycophenolate	2 (8.7)
Cyclophosphamide	4 (17.4)
Rituximab	3 (13)
Intravenous immunoglobulins	13 (56.5)
Two combined drugs	12 (52.2)
Three or more combined drugs	9 (39.1)
Response to PE	
Complete disease remission	10 (43.5)
Clinical improvement (any)	10 (43.5)
Null response	2 (8.7)
Not available	1 (4.3)
Adverse effects after PE	
Yes/No	2 (8.7)/21 (91.3)
Allergic	1 (4.3)
Vascular	1 (4.3)
Relapses after PE	
Yes/No/Unknown	
Adults	4 (20)/13 (65)/3 (15)
Under 18 years old	0/3 (100)/0
Disease-free interval in months, mean; SD	76; 83.5
Number of relapses, mean; SD	3.25; 1.9
Time until remission, months; SD	31.6; 63
Deaths	3 (13)

Abbreviations: PE, plasma exchange; SD, standard deviation.

**Table 2:** Clinical response after plasma exchange of available anti-SRP patients included in this review.

	Before PE	After PE		
	n/N (%)	n/N (%)	*p* Value	Response Rate (%)
Clinical manifestations				
Muscle weakness	20/20 (100)	9/20 (45)	0.001	11/20 (55)
Myalgia	3/12 (25)	0/12 (0)	0.250	3/3 (100)
Dysphagia	5/12 (41.7)	0/12 (0)	0.063	5/5 (100)
Anorexia	1/12 (8.3)	0/12 (0)	1	1/1 (100)
Dyspnea	3/12 (25)	0/12 (0)	0.250	3/3 (100)
Asthenia	2/12 (16.7)	0/12 (0)	0.500	2/2 (100)

Abbreviations: PE, plasma exchange. The percentage was calculated according to available data. Comparisons were performed using McNemar’s test.

**Table 3:** Treatments used before and after plasma exchange in patients with available information.

	Before PE	After PE	
	n/N (%)	n/N (%)	*p* Value
Corticosteroids	10/11 (90.9)	10/11 (90.9)	1
Pulsed corticosteroids	3/11 (27.3)	0/11	0.250
Additional immunosuppressant	11/13 (84.6)	10/13 (76.9)	1
Corticosteroids sparing agents	5/12 (41.7)	7/12 (58.3)	0.687
Methotrexate	2/11 (18.2)	4/11 (36.4)	0.500
Tacrolimus	0/12 (0)	0/12 (0)	1
Ciclosporin	1/12 (8.3)	1/12 (8.3)	1
Mofetil mycophenolate	2/12 (16.7)	1/12 (8.3)	1
Cyclophosphamide	3/12 (25.0)	2/12 (16.7)	1
Azathioprine	4/11 (36.4)	3/11 (27.3)	1
Rituximab	3/13 (23.1)	3/13 (23.1)	1
Intravenous immunoglobulins	7/12 (58.3)	0/12 (0)	0.016

Abbreviations: PE, plasma exchange. Percentages were calculated according to available data. Comparisons were performed using McNemar’s test.

## Data Availability

No new data have been reported.
